# Effects of Different Nitrogen Forms on Blackberry Fruit Quality

**DOI:** 10.3390/foods12122318

**Published:** 2023-06-08

**Authors:** Yongkang Duan, Haiyan Yang, Zhiwen Wei, Hao Yang, Sufan Fan, Wenlong Wu, Lianfei Lyu, Weilin Li

**Affiliations:** 1 Institute of Botany, Jiangsu Province and Chinese Academy of Sciences (Nanjing Botanical Garden Mem. Sun Yat-Sen), Jiangsu Key Laboratory for the Research and Utilization of Plant Resources, Nanjing 210014, China; dyk@njfu.edu.cn (Y.D.); fanfan19951211@163.com (S.F.); 1964wwl@163.com (W.W.); njbglq@163.com (L.L.); 2 Co-Innovation Center for Sustainable Forestry in Southern China, College of Forestry, Nanjing Forestry University, Nanjing 210037, China; wzw0709wzw@163.com (Z.W.); yanghao_19940720@163.com (H.Y.)

**Keywords:** blackberry, nitrogen, fruit quality, active substances, antioxidant activity

## Abstract

To study the optimal form of nitrogen (N) application and to determine the best harvest date for blackberries, different N fertilizers were applied during the critical growth period of blackberry plants. The results showed that NH_4_^+^–N significantly improved the appearance of blackberry fruits, including their size, firmness, and color, and promoted the accumulation of soluble solids, sugars, anthocyanin, ellagic acid, and vitamin C (VC), while fruit treated with NO_3_^−^–N accumulated more flavonoids and organic acids and had improved antioxidant capacity. In addition, the fruit size, firmness, and color brightness decreased with the harvest period. While the contents of sugars, anthocyanin, ellagic acid, flavonoids, and VC were higher in the early harvests and then decreased as the season progressed, the total antioxidant capacity and DPPH radical scavenging capacity increased. In all, application of NH_4_^+^–N is recommended, as it is more beneficial to fruit appearance, taste, and nutritional quality. Harvests in the early stage help to obtain a good fruit appearance, while harvests in the middle and later stages are more beneficial to fruit taste and quality. This study may help growers to determine the best fertilization scheme for blackberries and choose the appropriate harvest time according to their needs.

## 1. Introduction

Blackberries (*Rubus* spp.) are native to Europe and America, and are widely cultivated in warm regions of the world, mainly in North America, Europe, Asia, and South America. Blackberry fruits are rich in sugars, VC, superoxide dismutase (SOD), anthocyanins, ellagic acid, flavonoids, carotenoids, amino acids, and other mineral nutrients [1]. Because of their rich nutrition and unique taste, they have been recommended as the third generation of emerging small berries by FAO [2]. Souza et al. [3] found that blackberries had the highest antioxidant activity and phenol and anthocyanin content compared to other berries such as red raspberries, strawberries, cherries, and blueberries, even among different varieties. In addition, epidemiological and clinical studies have shown that blackberries have anti-inflammatory, bactericidal, and anti-aging properties, and that long-term consumption of blackberries may help to enhance immunity, prevent obesity, and reduce the risk of neurodegenerative diseases and various cancers [4,5].

Blackberries can be sold as fresh fruit, but they are highly perishable due to their high respiration rate and delicate skin. Therefore, they are often processed into jam, juice, yogurt, fruit wine, and pastries, and can also be made into dyeing agents [6]. The quality of fruit is extremely important for consumers and food processors, and high-quality fruits tend to have higher market potential. Therefore, growers all over the world want berries with high firmness, large size, good taste, and rich nutrients. These wishes can be realized through management of cultivation techniques, including fertilization. 

Blackberry growers often use large amounts of N fertilizer to improve yield and quality. However, N use efficiency is very low, and less than 40% of the N can be directly absorbed by crops [7]. Simply increasing the use of N fertilizer not only fails to bring high economic benefits, but also leads to imbalances in plant nutrition. In addition, excess nutrients are lost to the environment, resulting in the destruction of soil and water resources. Ammonium (NH_4_^+^) and nitrate (NO_3_^−^) are the two main N forms absorbed from soil and utilized by plants [8]. In addition, plants can also absorb organic N forms including amino acids and urea. Urea once became the most widely used organic N form in global agriculture due to its advantages of high N content and easy transportation [9]. At micromolar concentrations, most plants prefer to absorb NH_4_^+^ rather than NO_3_^−^ [10] because the energy cost associated with the assimilation of NH_4_^+^ is lower than that of NO_3_^−^ [8]. Most plants are sensitive to NH_4_^+^, and NH_4_^+^ is usually toxic to plants at millimole concentrations [11]. Some researchers believe that NH_4_^+^ can easily cause imbalances in hormone homeostasis in plants and lead to reductions in photophosphorylation [12,13]. In contrast, NO_3_^−^ can be stored in cells at high concentrations [14]. Most plants grow better in soils that contain mainly NO_3_^−^, while only a few plants prefer NH_4_^+^, such as rice, blueberries, and tea trees [15,16,17]. 

N not only affects the vegetative growth of plants, but also plays an important role in regulating fruit quality [2,18]. Yang et al. [2] found that rational application of N fertilizer could significantly promote photosynthesis in blackberry plants and increase fruit yields and anthocyanin, polyphenol, and ellagic acid contents. Ali et al. [18] observed that when N fertilizer application increased from 60 kg ha^−1^ to 100 kg ha^−1^, the pH value and soluble solid, sugar, and anthocyanin contents of blackberry fruits increased significantly. Edgley et al. [19] also found that high N treatment can significantly improve the biomass and yield of blackberry fruits in the same year, while fruit quality significantly decreases after the second year. In addition, N forms also affected fruit growth and development. In apples, increasing the proportion of NH_4_^+^ significantly reduced the fruit hardness and increased the N and K contents in the fruit [20]. Compared with using NO_3_^−^ as the only N source, increasing NH_4_^+^ in the growth medium by 25% can increase the yield of tomatoes [21]. Shi et al. [22] found that increasing the proportion of NH_4_^+^ in nutrient solutions reduced the number of flowering strawberries but did not affect the vegetative growth of strawberries. However, unreasonable application of N fertilizer could reduce the nutritional quality and storage life of certain berries, such as blackberries [23], strawberries [24], and grapes [25]. For example, it has been reported that excessive application of NO_3_^−^ can reduce the quality of berries [26].

Previous studies showed that blackberry plants fed with NH_4_^+^ had higher biomass and chlorophyll contents and stronger antioxidant systems, so it was thought that blackberry plants preferred to absorb NH_4_^+^ [27,28]. However, this conclusion needs to be improved. People tend to pay more attention to blackberries’ appearance and nutritional quality. Although good vegetative growth is a prerequisite for the formation of high-quality fruit, excessive stimulation of vegetative growth may also lead to poor fruit quality. For example, high N availability can promote the vegetative growth of tomatoes, leading to the preferential distribution of excessive nutrients to leaves and branches, resulting in low fruit setting rates and reduced sugar contents [29]. There were few reports on the improvement of fruit quality through the use of different types of N fertilizer. In addition, the effects of different N fertilizers on the regulation mechanisms of various secondary metabolites in blackberry fruits are still unclear.

The purpose of this study is to determine the optimal N application type for blackberry plants and to determine the most suitable harvest date. Our results are of great significance for improving blackberry fruit quality and reducing production costs and environmental pollution.

## 2. Materials and Methods

### 2.1. Plant Materials and Experimental Design

The experiment was conducted in a greenhouse at the Institute of Botany, Chinese Academy of Sciences of Jiangsu Province from March to July 2022. The experimental materials were 2-year-old plants of the blackberry cultivar ‘Ningzhi 4’ (Kiowa × Hull). The plants selected for transplanting into 23 L containers had uniform, healthy growth and no pests or sicknesses. The pots were filled with a soilless substrate (pH 4.8) consisting of coconut coir, charcoal, and perlite in a 4:3:1 ratio, followed by 20 d of fertilizer control (distilled water only) to deplete the residual nutrients in the plants and make them more sensitive to fertilization. During the experiment, blackberry plants were divided into four groups including a no-N fertilization group (CK), an NH_4_^+^–N group, an NO_3_^−^–N group, and a urea group. The fertilizer application formulations are shown in Table 1. To prevent nitrification by ammonium and urea, the stopper dicyandiamide was added to the substrate. With 18 pots per treatment, the experiment was conducted according to a completely randomized design. Fertilizer was applied twice a week, using 600 mL each time for 60 consecutive days of treatment. The total amount of N applied to each blackberry plant was kept consistent except for the control group. To make sure of fruit quality, three fruiting branches were left on each plant. In summer, new annual branches (basal branches) were promptly topped and lateral branches were shortened to hold back plant growth and ensure the best fruit quality. Since blackberries ripened continuously, they were picked weekly, on 6 June, 13 June, 21 June, and 28 June, respectively, when the black, glossy, and fully ripe fruits were picked for later experimental index measurements.

### 2.2. Measurement of Fruit Shape Index, Soluble Solids and Acid Content

Fruit shape indicators and soluble solid and acid contents were measured immediately after fresh fruit picking. Twenty mature blackberry fruits were randomly selected from plants from each treatment group for measurement. The transverse and longitudinal diameters of the fruits were measured with a digital vernier caliper (Cat. No. DL91150, Ningbo Deli Tools Co., Ltd., Ningbo, China) and weighed with an electronic balance (Cat. No. FA1004, Shanghai Sunyu Hengping Instruments Co., Ltd., Shanghai, China) with an accuracy of 0.01 g. Fruit hardness was measured using a fruit hardness tester (Cat. No. 9300 (KM-5), Kyoto, Japan). Fruit color was measured with a portable colorimeter (3NH SR-66; Shenzhen 3NH Technology Co., Ltd., Shenzhen, China). Color coordinates were recorded as L*, a*, and b* [2]. Soluble solids were measured using a pocket brix-acidity meter (Cat. No. 7100 (PAL-5), Atago Co., Ltd., Bellevue, WA, USA). The acid content was also determined through electrical conductivity by a pocket brix-acidity meter (Cat. No. 7100 (PAL-5), Atago Co., Ltd., Bellevue, WA, USA). Fifty grams of fresh blackberries were weighed and beaten into a homogenate under ice bath conditions. The blackberry homogenate was diluted with distilled water at a ratio of 1:50 before acid content determination, and the acid determined was the total acid content in the fruit. 

### 2.3. Measurement of Sugar Content

Samples were harvested and placed in dry ice and sent to the laboratory. Samples from all periods were refrigerated at −80 °C immediately after harvesting. Subsequent physiological indicators were measured uniformly to reduce experimental errors. First, ten ripe blackberries were selected and crushed into a uniform pulp using liquid nitrogen. Then, 3 g blackberry homogenate was put into a 50 mL centrifuge tube with 30 mL of distilled water for extraction. The extract was shaken for 30 min at 25 °C and then centrifuged at 5000 r∙min^−1^ for 10 min, after which the supernatant was taken for measurement. The content of fructose was determined using the Fructose Quantitative Test Kit (A085-1-1, Nanjing Jiancheng Bioengineering Institute, Nanjing, China). The standard curve was made before the determination, and was y = 3.739x + 0.0045 (R^2^ = 0.9998). The matrix solution was mixed well with the sample, and the reaction was carried out in boiling water for 8 min, followed by rapid cooling with cold water. The OD value was measured at 285 nm by spectrophotometer. The glucose content was determined with reference to the glucose oxidase method [30]. The sucrose content was also determined using a sucrose test kit (A099-1-1, Nanjing Jiancheng Bioengineering Institute, Nanjing, China), and the absorbance was calculated by measuring the maximum absorption peak (290 nm) of the product of sucrose in the hydrolysis solution after boiling in water at 100 °C. 

### 2.4. Determination of Antioxidant Capacity

Total antioxidant capacity (T-AOC) and 1,1-diphenyl-2-picrylhydrazyl radical (DPPH) scavenging capacity were determined using T-AOC (A015-3-1) and DPPH (A153-1-1) determination kits (Nanjing Jiancheng Institute of Bioengineering, Nanjing, China). The T-AOC was determined under acidic conditions, where the antioxidant substances could reduce Fe^3+^-TPTZ to Fe^2+^-TPTZ, which was indicated by a color change to blue. Next, 0.2 g of blackberry homogenate was weighed accurately, then 0.8 mL of physiological saline was added and mechanically ground into the homogenate under ice bath conditions. The supernatant was then centrifuged at 10,000 r∙min^−1^ for 5 min at 4 °C before being taken for measurement. Then, 5 µL of the supernatant was aspirated and mixed with 180 µL of FRAP working solution in a water bath at 37 ℃ for 37 min, and the absorbance was read at 593 nm. Finally, the total antioxidant capacity in the sample was calculated. The standard curve was y = 3.521x − 0.0578 (R^2^ = 0.9813). For the determination of the DPPH radical scavenging capacity, the sample was pretreated by weighing 0.2 g blackberry homogenate, adding 1 mL of 80% methanol solution, homogenizing on ice, centrifuging at 10,000 r∙min^−1^ for 10 min, and then removing the supernatant for measurement. The standard curve was y = 0.032x + 0.0094 (R^2^ = 0.9981), and an amount equivalent to the antioxidant trolox calculated from the standard curve was used to express the DPPH radical scavenging ability of the sample.

### 2.5. Determination of Vitamin C Content

The determination of vitamin C (VC) content was performed using a Vitamin C Assay Kit (A009-1-1, Nanjing Jiancheng Bioengineering Institute, Nanjing, China). This operation is based on the rapid interaction of Fe^3+^ with reduced ascorbic acid to form Fe^2+^, which then reacts with phenanthroline in a color development reaction. Next, 0.2 g blackberry homogenate was accurately weighed and added to saline at a mass to volume ratio of 1:9, then ground in an ice bath. Subsequently, the mixture was centrifuged at 5000 r∙min^−1^ for 10 min. Next, 0.15 mL of the supernatant was aspirated, added to 0.45 mL of the application solution, then vortexed, mixed, and centrifuged at 4000 r∙min^−1^ for 10 min. After standing for 15 min, the upper layer of clear liquid was the supernatant. Next, 0.4 mL of supernatant was aspirated, then reagent application solutions 2, 3, and 4 were added and mixed thoroughly for 30 min at 37 °C in a water bath. Finally, reagent 5 was added, mixed well, and left to stand for 10 min. The maximum absorption peak (536 nm) was used to determine the VC content in the sample.

### 2.6. Determination of Anthocyanin Content

The total anthocyanin content was determined by the pH differential method [31]. After weighing 50 g of fresh frozen blackberries and beating them into a homogenate in a grinder with liquid nitrogen, 3 g of the homogenate was moved into a 50 mL centrifuge tube and 30 mL of ethanol solution (containing 0.1% formic acid by volume) was added at a material ratio of 1:10, mixed thoroughly, and sonicated at 35 °C for 20 min at 60 Hz using a numerical control ultrasonic cleaner (KQ-300DE, Kunshan Ultrasonic Instruments Co., Ltd., Suzhou, China). The supernatant was centrifuged at 5000 r∙min^−1^ for 5 min and taken for measurement. A total of 300 µL of supernatant was added to 2.7 mL of pH 1.0 and pH 4.5 buffer, respectively, and the absorbance values were measured as 510 nm and 700 nm, respectively, using a UV-visible spectrophotometer (759S, Shanghai Jing Hua Technology Instruments Co., Ltd., Shanghai, China). Then, the total anthocyanin content in blackberry fruits was calculated.

### 2.7. Determination of Ellagic Acid Content

The determination of ellagic acid content in fruits was based on the method of Maas et al. [32] with slight modifications. First, 50 g of fresh frozen blackberries were weighed and pulsed in an ice bath. Next, 3 g of the pulsed mixture were taken and added to 30 mL of anhydrous ethanol after ultrasonic extraction at 80 °C for 20 min. The resulting mixture was then centrifuged at 6000 r∙min^−1^ at 25 °C for 10 min. Then, 1 mL of the supernatant was accurately measured and mixed with 4 mL of 0.1 mol∙L^−1^ NaOH solution, and the solution turned blue after 15 min of full reaction. The OD value was measured as 357 nm using a UV-visible spectrophotometer (759S, Shanghai Jing Hua Technology Instruments Co., Ltd., Shanghai, China), and the ellagic acid content was calculated corresponding to the standard curve, y = 0.0504x + 0.003 (R^2^ = 0.9996).

### 2.8. Determination of Total Phenol Content

The total phenol content was determined using the Folin–Ciocalteu method [33], the basis of which was slightly modified. First, 50 g of fresh frozen blackberries were weighed and pulsed with liquid nitrogen, and 3 g of the resulting homogenate were taken and added to 30 mL of 50% ethanol solution. Then, the supernatant was obtained by centrifugation after 20 min of ultrasonic extraction at 60 Hz at 35 °C. The supernatant was diluted 5 times, then 1 mL was added to 0.5 mL of Folin’s reagent and mixed well. Next, 2 mL of 7.5% saturated sodium carbonate solution was added. Finally, 6.5 mL of distilled water was added, vortexed, and shaken for 5 min, then shaded and left to stand for 2 h. The absorbance value was measured as 765 nm using a UV-visible spectrophotometer (759S, Shanghai Jing Hua Technology Instruments Co., Ltd., Shanghai, China). The total phenol content was calculated using the corresponding standard curve, y = 0.12839x + 0.00045 (R^2^ = 0.9994).

### 2.9. Determination of Flavonoid Content

The total flavonoid content was determined according to the GBT 20574-2006 national standard. Samples were ground to a powder with liquid nitrogen. Then, 3 g of the samples were extracted in 30 mL of 95% ethanol for 45 min at 65 °C. Next, 3 mL of supernatant were mixed with 10 mL of 95% ethanol, 1 mL of 100 g/L Al(NO_3_)_3_, and 1 mL of 9.8 g/L CH_3_COOK in a 50 mL volumetric flask. The solution was adjusted to 50 mL with deionized water, left to stand for 1 h at room temperature, and the OD value was measured as 415 nm using a UV-visible spectrophotometer (759S, Shanghai Jing Hua Technology Instruments Co., Ltd., Shanghai, China). The flavonoid content was calculated according to the corresponding standard curve, y = 0.0273x + 0.0409 (R^2^ = 0.9994). The flavonoid content of the blackberries was expressed as milligams per gram of FW (mg/g FW).

### 2.10. Statistical Analysis

Statistical analysis was performed using IBM SPSS Statistics 25 software (IBM Corp., Armonk, NY, USA). Means were compared using one-way ANOVA, and Duncan’s comparison method was used for multiple comparisons, with significant differences at *p* < 0.05.

## 3. Results

### 3.1. Fruit Size, Weight and Firmness

**Figure 1:**
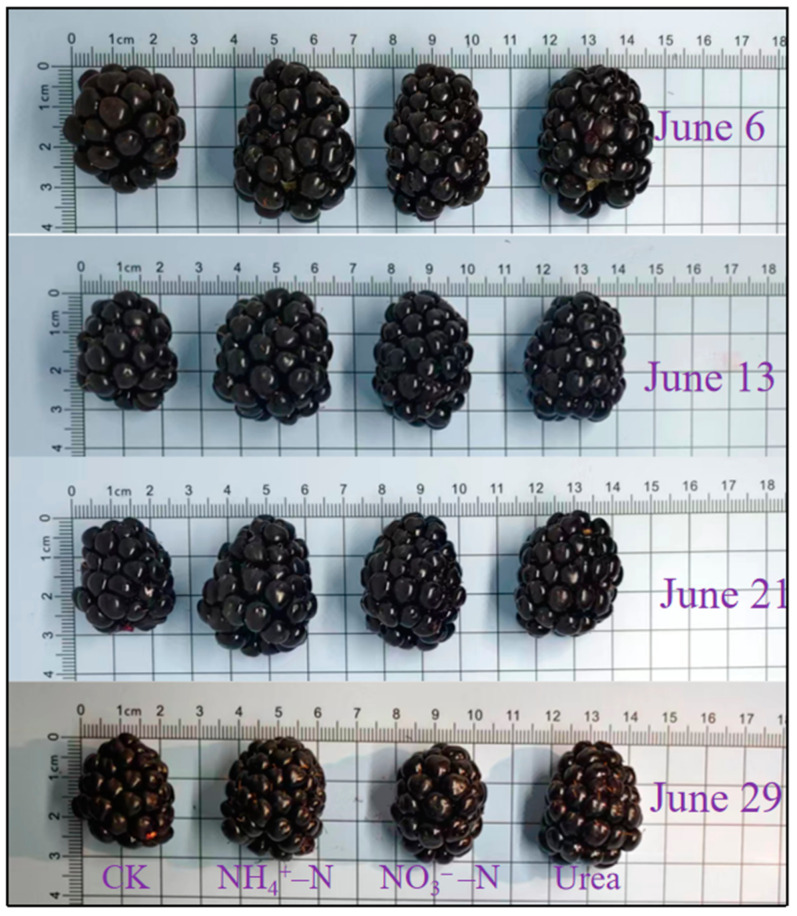
Morphological characteristics of ripe blackberry fruits at different harvest dates under different N treatments.

Different N fertilizer treatments had significant effects on the horizontal diameter, longitudinal diameter, fruit weight, and firmness of ripe blackberry fruits (Table 2). Compared to the fertilizer treatment, the no-N fertilizer (CK) condition had the smallest (*p* < 0.05) horizontal diameter, longitudinal diameter, fruit weight, and firmness. Overall, horizontal diameter, longitudinal diameter and fruit weight were significantly higher under the NH_4_^+^–N and urea treatments compared to the CK and NO_3_^−^–N treatments (Figure 1, Table 2). At the end of the harvest season (S4), fruit weight reached its lowest value, decreasing by 17.39% compared to the highest value (8.97 g). The fruit firmness was highest under the NH_4_^+^–N treatment. Overall, fruit firmness and volume were greatest at the beginning of the harvest season (S1) and then gradually decreased.

### 3.2. Fruit Soluble Solids, Acid, and Color

During the determination of the total soluble solid (TSS) content of the fruit, we found that NO_3_^−^–N treatment significantly reduced the TSS content of the fruit, while NH_4_^+^–N significantly promoted the accumulation of TSS (Table 3). The TSS content was influenced by the harvest date and showed a tendency to increase and then decrease with the extension of the harvesting season, reaching its highest value (12.17) during the S3 (21 June) period. The acid content of blackberry fruits also differed significantly (*p* < 0.05) under different N treatments. Unlike other fruits, the acid content of blackberry fruits was quite high, ranging from 1.33% to 1.91% (Table 3). The acid content in the fruits was higher under NO_3_^−^–N and urea treatments compared to the NH_4_^+^–N treatment, but the relative differences between NO_3_^−^–N and urea were influenced by the harvest season. The acid content showed a trend of decreasing and then increasing with the extension of the harvesting season. The L* color values were between 11.48 and 17.72, the a* values were between 0.10 and 0.42, and the b* values were between −2.13 and 3.46 for blackberries. The measured parameters, L*, a*, and b*, did not differ significantly between N treatments, and only L* values differed between sampling periods (*p* < 0.05), with the largest L* values being recorded during the S1 and S2 terms.

### 3.3. The Content of Sugars

**Figure 2:**
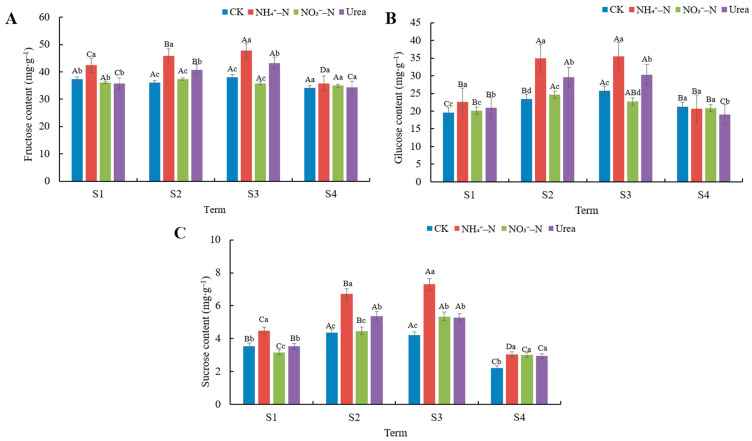
Variation in fruit sugar content in different harvest periods under different N treatments. (**A**) Fructose content, (**B**) glucose content, (**C**) sucrose content. The data shown are the averages ± SDs (*n* = 3). Different capital letters indicate that there are significant differences between different harvest periods under the same treatment. Different lowercase letters indicate that there are significant differences among different N treatments in the same harvest period (*p* < 0.05).

We systematically studied the differences in the sugar content of blackberry fruits harvested at different dates under different N treatments. The results revealed that the changes in the sugar content of blackberry fruits were similar to the results for soluble solids. Overall, the fructose, sucrose, and glucose contents accumulated in fruits under the NH_4_^+^–N treatment were the highest, followed by those under the urea treatment (Figure 2). The absence of N fertilization seemed to be detrimental to the accumulation of sugars in the fruits. In addition, fructose underwent the largest increase in mature fruits, followed by glucose and sucrose. Among the three sugars measured, fructose and glucose were the main sugars accumulated in blackberry fruits, accounting for about 90% of the total sugars. The changes in the content of the three sugars showed the same trend, and they all showed a trend of increasing and then decreasing, with the highest sugar content in blackberry fruits in S3 (21 June) and the lowest in S4 (29 June). The fructose content of blackberry fruit ranged from 34.13 to 47.80 mg∙g^−1^ FW, the glucose content ranged from 19.01 to 35.50 mg∙g^−1^ FW, and the sucrose content ranged from 2.22 to 7.29 mg∙g^−1^ FW.

### 3.4. Active Antioxidant Substance Content Analysis

**Figure 3:**
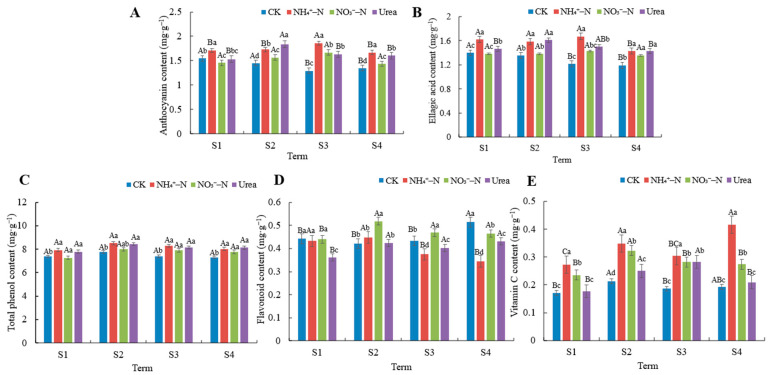
Variation in the active antioxidant substance contents of fruit in different harvest periods under different N treatments. (**A**) Anthocyanin content, (**B**) ellagic acid content, (**C**) total phenol content, (**D**) flavonoid content, (**E**) vitamin C content. The data shown are the averages ±SDs (n = 3). Different capital letters indicate that there are significant differences in different harvest periods under the same treatment. Different lowercase letters indicate that there are significant differences among different N treatments in the same harvest period (*p* < 0.05).

Importantly, blackberry fruits contain a variety of naturally occurring active antioxidant substances. We found some differences in the anthocyanin, ellagic acid, polyphenol, flavonoid, and vitamin C contents of the fruits under different N treatments (Figure 3). In general, the anthocyanin and ellagic acid contents were higher under NH_4_^+^–N and urea treatments than in NO_3_^−^–N and CK treatments, and the lowest content was recorded for the no-nitrogen condition (CK). The anthocyanin and ellagic acid contents showed slight variations depending on the harvest period and were highest during the S3 term. N fertilization significantly increased the total phenol content in the fruits (*p* < 0.05); however, there seemed to be no significant difference in the accumulation of polyphenols among the different N forms, and the total phenol content remained around 8 mg∙g^−1^ FW with the changes in the harvest period. Notably, the NO_3_^−^–N treatment was more favorable to flavonoid accumulation compared to the NH_4_^+^–N and urea treatments. The highest value of flavonoid content (0.52 mg∙g^−1^ FW) was reached during the S2 term, and increased by 15.56% and 23.81% relative to the NH_4_^+^–N and urea treatments. Interestingly, the CK treatment also favored flavonoid accumulation in the fruits. The effects of different N treatments on the accumulation of VC content in the fruits were ranked as follows: NH_4_^+^–N > NO_3_^−^–N > urea > CK (*p* < 0.05). A mid-late harvest date seemed to be more favorable for obtaining blackberry fruits with high VC content.

### 3.5. Antioxidant Ability Analysis

**Figure 4:**
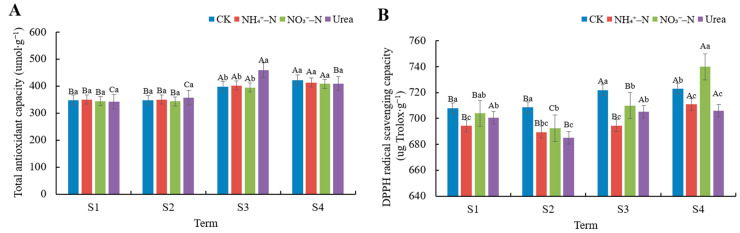
Variation in the antioxidant capacity of fruit in different harvest periods under different N treatments. (**A**) Total antioxidant capacity, (**B**) DPPH radical scavenging capacity. The data shown are the averages ±SDs (n = 3). Different capital letters indicate that there are significant differences in different harvest periods under the same treatment. Different lowercase letters indicate that there are significant differences among different N treatments in the same harvest period (*p* < 0.05).

We used the total antioxidant capacity and DPPH radical scavenging capacity to evaluate the fruit antioxidant capacity under different N treatments at different harvest dates. In the S3 term, the highest total antioxidant capacity was observed in urea treated fruits, but in the S1, S2, and S4 terms, the total antioxidant capacity of fruits under different N treatments did not show significant differences (Figure 4A). Therefore, we could not conclude whether the different N forms had an effect on the total antioxidant capacity of the fruits. However, it is certain that the total antioxidant capacity of blackberries was increased in the late-harvest S3 and S4 terms. Different N treatments had a significant effect on the DPPH radical scavenging capacity of blackberry fruits (*p* < 0.05, Figure 4B). NO_3_^−^–N significantly increased the DPPH radical scavenging capacity in fruits, reaching the highest value of 739.81 ug Trolox∙g^−1^ FW in the S4 term (*p* < 0.05). In addition, The CK treatment also stimulated the DPPH radical scavenging capacity of the fruits, reaching the highest values of 708.00 ug Trolox∙g^−1^ FW, 708.57 ug Trolox∙g^−1^ FW, and 721.99 ug Trolox∙g^−1^ FW in the S1, S2, and S3 terms, respectively. Similarly, the DPPH radical scavenging capacity of the fruits was significantly increased in the S3 and S4 terms during the late harvest season.

### 3.6. Correlation and PCA of Physiological and Quality Indexes

**Figure 5:**
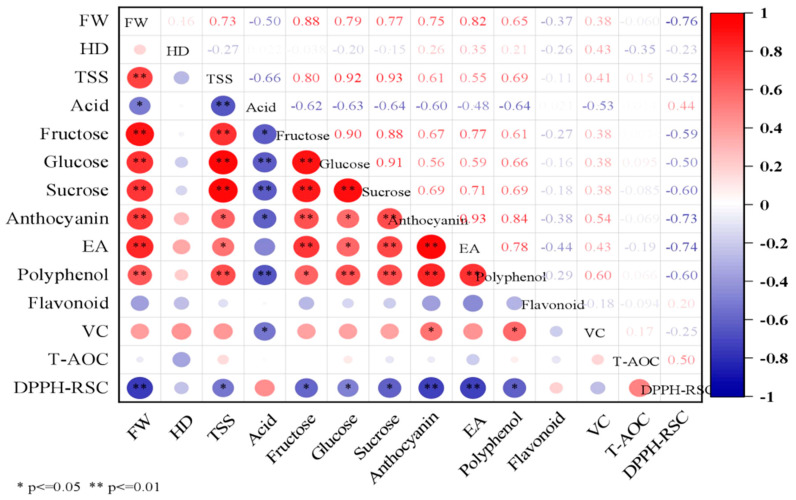
Correlation matrix of physiological indexes of blackberry fruits. The results were analyzed through Pearson correlation analysis. * represents a significant correlation at the 0.05 level, and ** represents a significant correlation at the 0.01 level. FW, fruit weight; HD, hardness; TSS, total soluble solids; EA, ellagic acid; VC, vitamin C; T-AOC, total antioxidant capacity; DPPH-RSC, DPPH radical scavenging capacity. The same as follows.

The results of the correlation matrix analysis (Figure 5) showed that fruit weight under different N treatments was highly significantly positively correlated with the contents of TSS, fructose, glucose, sucrose, anthocyanin, ellagic acid, and polyphenol accumulated in the fruits (*p* < 0.01), with correlation coefficients of 0.73, 0.88, 0.79, 0.77, 0.75, 0.82, and 0.65, respectively. Fruit weight was negatively correlated with acid content (*p* < 0.05) and DPPH radical scavenging capacity (*p* < 0.01), with correlation coefficients of −0.50 and −0.76, respectively. TSS content in fruit was highly significantly positively correlated with fructose, glucose, sucrose, and polyphenol contents (*p* < 0.01), with correlation coefficients of 0.80, 0.92, 0.93, and 0.69, respectively; additionally, TSS content was negatively correlated with acid content (*p* < 0.01) and DPPH radical scavenging capacity (*p* < 0.05), with correlation coefficients of −0.66 and −0.52, respectively. The levels of VC were significantly positively correlated with anthocyanin and polyphenol contents (*p* < 0.05), with correlation coefficients of 0.54 and 0.60, respectively, and VC levels were negatively correlated with acid content, with a correlation coefficient of −0.53. The levels of polyphenols, ellagic acid, and anthocyanin in blackberry fruits were significantly positively correlated with sugar content and fruit weight (*p* < 0.05). In conclusion, the above analysis showed that blackberries respond to different N fertilization by regulating fruit size, antioxidant capacity, and fruit quality.

We performed principal component analysis (PCA) on various physiological indicators, which resulted in the isolation of 14 components, among which the first, second, and third principal components (eigenvalues > 1) explained 54.79%, 14.59%, and 9.98% of the variance in the data, respectively, with a total contribution of 79.36% (>75%) (Table 4). Thus, the three principal components adequately covered the information of the 14 physiological indicators with good data interpretation. Fruit weight, TSS, fructose, sucrose, glucose, anthocyanin, polyphenols, and ellagic acid were significantly positively correlated with PC1, while acid and DPPH free radical scavenging capacity were significantly negatively correlated with PC1 (Table 5). Fruit firmness was significantly negatively correlated with PC2, and the total antioxidant capacity was significantly positively correlated with PC3. 

## 4. Discussion

Fertilizers play a very important role in deciding the appearance, taste, nutrient content, and other quality parameters of horticultural crops. N fertilizer, especially, is one of the nutrients most demanded by plants during their lifetime. N availability is extremely important for plant growth and development because it is not only an important component of many living substances such as nucleic acids, enzymes, ATP, and proteins, but also maintains the structure and function of chloroplasts [34]. Size and firmness are very important physical traits of blackberry fruits, and fruits with high firmness are less likely to undergo rot and spoilage and are more suited to long-distance transport and extended storage periods. Generally speaking, blackberries with high firmness and large size are more likely to be preferred by consumers. Our study found that blackberry fruits had the largest transverse diameter, longitudinal diameter, fruit weight, and firmness when treated with NH_4_^+^–N or urea (Table 2, Figure 1). In contrast, these four indicators were minimal when NO_3_^−^–N was used as the sole N source. This suggested that NH_4_^+^–N is beneficial in increasing the size and firmness of blackberry fruits. In contrast to the apple study, an increase in the NH_4_^+^/NO_3_^−^ ratio did not change fruit size, but significantly reduced fruit firmness [20]. In general, increasing the level of NH_4_^+^–N in the nutrient medium decreases the fruit firmness. Ca is a constituent of the cell wall and is important in regulating the maintenance of the integrity of the membrane system, and high concentrations of NH_4_^+^–N reduce Ca^2+^ uptake by the roots, which leads to a decrease in Ca content in the fruits and shortens the postharvest life [20,35,36]. Our findings show that the regulation of fruit firmness is extremely complex and is influenced by various factors such as the species itself, temperature at harvest, humidity, and cultivation techniques. Furthermore, in our previous study using X-ray energy spectroscopy analysis, we found that Ca levels in leaves were significantly higher in NH_4_^+^–N fed blackberry plants than in those under NO_3_^−^–N treatment [27]. Therefore, it could not be determined whether NH_4_^+^–N would reduce Ca levels in the fruits. The mechanism by which NH_4_^+^–N increases fruit firmness is currently unclear. 

Overall, blackberry fruit size (including fruit horizontal diameter and longitudinal diameter) and fruit weight showed a gradual decrease with the harvest time (Table 1). In most cases, it is a very common phenomenon that fruit volume becomes smaller approaching the end of the harvesting season [19,37]. Mikulic-Petkovsek et al. [6] also found that near the end of the harvesting season the smallest fruit weight was observed when they studied the fruit quality characteristics of different cultivars of blackberry during each harvesting period. The decrease in berry size and weight may be due to a gradual increase in fruit transpiration and impeded transport of nutrients in the bast as the harvesting season extends [38]. It could also be ascribed to carbohydrate competition [39], since the sugar content in the fruits was lowest at the end of the harvest season (Figure 2), while fruit weight was positively strongly related with fructose, glucose, and sucrose (Figure 5). In addition, at the end of the harvest season, the fruits gradually become softer, reaching a minimum value of 0.37 kg·cm^−2^ in the S4 term. This was consistent with the findings of Fernandez-Salvador et al. [37] and Edgley et al. [19], who both reported a gradual decrease in firmness of blackberry fruits at the end of the harvesting period. In conclusion, in production practices where the production target is large fruit with high firmness, NH_4_^+^–N or urea application should be selected, and it is more desirable to harvest the fruits in the first harvest period after blackberry ripening. 

The first sight that consumers see when purchasing fruits is the color of the fruits. Blackberries that are black and shiny in appearance are more popular with consumers because dull color is associated with lack of freshness and over-ripeness [40,41]. Our results showed that blackberry fruits had the largest L* values in the S1 and S2 terms, where the larger L* values indicated brighter fruit surfaces and greater attractiveness to consumers; therefore, picking blackberries in the early harvest period is beneficial to improving fruit brightness. L*, a*, and b* values did not differ significantly between N treatments, indicating that N forms do not seem to have significant effects on fruit color. This is because fruit color is the result of a confluence of various factors, including variety, soil, humidity, temperature, cultivation techniques, and harvest time [42]. 

Flavor is one of the most important criteria by which consumers measure fruit quality, and although good appearance (color, size, and firmness) is important, consumers will choose to buy again only when they are very satisfied with the taste of the fruit. Sweetness is usually related to soluble solids and sugar content, and acidity is mainly related to citric and malic acid content, which in turn determine the fruit quality and flavor [36]. NH_4_^+^–N significantly increased the soluble solid content in the fruits (Table 3), which was mainly related to the higher sugar content under NH_4_^+^–N treatment (Figure 2). The correlation analysis showed a highly significant positive correlation between soluble solid content and sugar content in the fruits (Figure 5). In this study, it was found that N fertilizer application seemed to stimulate sugar transport to the berries. When NH_4_^+^–N was applied, the highest values of fructose, glucose and sucrose contents were found in blackberry fruits. In contrast, the lowest sugar content was found in berries under the CK treatment. Carbon and N metabolism are mutually regulating and interacting in plants [43]. The supply of NH_4_^+^–N resulted in the accumulation of more carbohydrates in the fruits, due in large part to the large carbon skeleton required for NH_4_^+^–N assimilation [44]. Similarly, the carbon assimilation process is dependent on the various enzymes and proteins produced by ammonium assimilation. NH_4_^+^–N has been previously been reported to promote photosynthesis in blackberry plants by increasing the N and chlorophyll content in the leaves. Conversely, N-deficient (CK) plants exhibited yellowing of leaves and thin plants [27]. Therefore, high photosynthesis is usually very beneficial for blackberry plants. On the one hand, the supply of NH_4_^+^–N accelerates the uptake, transport, and assimilation of N by the plants, promotes N metabolism, and accelerates the N uptake and utilization efficiency of the roots. On the other hand, NH_4_^+^–N is beneficial to photosynthesis, thus facilitating the synthesis of more sugars stored in the fruits, while these soluble sugars are good osmoregulatory substances that play a key role in maintaining cellular homeostasis and help alleviate the toxic effects of NH_4_^+^–N. It is notable that fructose and glucose contents were highest in blackberries at all stages of maturity, accounting for more than 90% of total sugars, regardless of the applied type of N fertilizer, which is consistent with the results of Kafkas et al. [45], who showed that blackberries mainly accumulate fructose and glucose. In general, fructose is sweeter than glucose and sucrose, and higher accumulation of fructose in fruits is desirable because sweeter fruits are usually more popular among consumers. In addition, NO_3_^−^–N is beneficial in increasing the total acid content in fruits. In a study on strawberries, it was found that the NH_4_^+^ to NO_3_^−^ ratio had little effect on the pH of the juice, but the acidity of the juice increased when a higher proportion of NO_3_^−^ was supplied [24]. Sernal et al. [46] found that when NO_3_^−^ was used as the sole N source, it significantly increased the acidity of ripe tomato fruits and increased the citric and malic acid contents. This suggests that NO_3_^−^–N caused poorer taste in blackberry fruits, while the fruits were sweeter and tasted better under NH_4_^+^–N treatment. In addition, the highest soluble solid and sugar contents of the fruits were found during the S3 term (21 June), and the lowest total acid contents of the fruits were found in the S2 and S3 terms; therefore, ripe blackberries could be selected for picking in the middle and late stages, which could lead to the best taste. If you want to select for brighter color, blackberries can be picked in the early stage of fruit ripening. 

Environmental conditions, fertilization type, and harvest period have been shown to affect the transport and distribution of nutrients by the plant, thus affecting the content and composition of secondary metabolites in the fruits, such as anthocyanins, ellagic acid, VC, flavonoids, and phenolic compounds [6,40,47]. These secondary metabolites enable blackberries to have extremely high antioxidant activity, which, unlike enzyme systems, can be ingested by the body; therefore, blackberries have the title of the king of antioxidants. It has been believed that high N availability reduces the content of antioxidants such as anthocyanins and VC in blackberries because it stimulates the nutritional growth of the plant, thus preferentially allocating resources to nutritional growth rather than reproductive growth and failing to increase secondary metabolites in the fruits [23,48]. However, different scholars have come up with different results [48]. Our study showed N fertilization, regardless of the type, increased the content of antioxidant substances in the fruits, except for flavonoids, while the N deficiency (CK) treatment decreased the antioxidant substances content. In a study of other plants, Chatzigianni et al. [49] similarly found that N fertilization significantly increased the total phenol content. In contrast, NH_4_^+^–N had an important role in promoting the accumulation of anthocyanin, ellagic acid, and VC. The effect of different N forms did not seem to differ significantly in terms of the total phenol content, which was maintained around 8 mg∙g^−1^ FW, in agreement with the levels reported by Kaume et al. (1.14 to 10.56 mg∙g^−1^ FW) [5]. Anthocyanins are the most important phenolic compounds in blackberries, accounting for about more than 50% of the phenolic compounds, and their extremely high anthocyanin content is the main cause of black color formation in blackberries [6]. Our study found that the anthocyanin content tended to increase as the ripening process of blackberries proceeded and began to decrease in the S3 term, although it has been reported that the anthocyanin content in blackberries increases continuously with the ripening process of the fruits [41]. Flavonoids are not only a nutritional component, but also have a function in regulating plant defense systems and signal transduction in response to biotic and abiotic stresses [50]. Notably, our results showed that NO_3_^−^–N and CK treatments significantly promoted the accumulation of flavonoids in fruits. It has been reported that NO_3_^−^–N and CK-treated blackberry plants exhibit toxicities compared to NH_4_^+^–N treated plants, and that the accumulation of flavonoids in the leaves facilitates resistance to this adversity, thus accelerating the flow of N to C metabolism [28]. Although we were not able to observe whether NO_3_^−^–N produced stress in fruits, the high accumulation of flavonoids is likely to be associated with a significant up-regulation of genes related to flavonoid synthesis in fruits, which requires further experimental evidence. In an experimental investigation of raspberries native to China, Fu et al. [51] found higher levels of flavonoids in smaller fruits. Lugaresi et al. [52] similarly found that blackberries with smaller fruits accumulated more flavonoids. This is similar to our findings that blackberry fruits under NO_3_^−^–N and CK treatments had smaller size and fruit weight. In conclusion, NH_4_^+^–N seemed to stimulate the accumulation of secondary metabolites such as anthocyanin, ellagic acid, and vitamin C in blackberry fruits, while NO_3_^−^–N favored the synthesis of flavonoids. 

High antioxidant activity is the most interesting property affecting the nutritional value of blackberry fruit. In our study, different N forms did not significantly change the total antioxidant capacity of the fruits. However, the total antioxidant capacity of blackberries was increased in late harvests during the S3 and S4 term. Most people believed that there was a correlation between total phenol content and antioxidant activity. However, we found no correlation through the correlation matrix (Figure 5). Similarly, Kähkönen et al. [53] did not find any correlation between them when they studied some berries, fruits, and vegetables. The total antioxidant activity is influenced by various aspects, not only as a result of anthocyanins, VC, flavonoids and phenolics, but also other secondary metabolites (carotenoids) and enzyme systems (SOD, peroxidase). The decrease in fruit quality is associated with the accumulation of reactive oxygen species, including O_2_−, H_2_O_2_, and OH^∙^, which damage the structure and function of cells by causing lipid peroxidation [54]. Our study found that NO_3_^−^–N and CK stimulate DPPH radical scavenging in fruits, which is important for maintaining intracellular free radicals at normal levels and enhancing the stability of the cell membrane system. The highest total antioxidant and DPPH free radical scavenging capacity of blackberries was observed in late harvests during the S3 and S4 terms. It can be concluded that harvesting ripe fruits in the middle and late stages seems to be more beneficial to increasing the antioxidant activity of the fruits.

## 5. Conclusions

Different N form treatments were applied during blackberry fruit development, and it was found that NH_4_^+^–N (or urea) significantly increased the size, firmness, and color and sugar, anthocyanin, ellagic acid, and VC contents of blackberry fruits, while NO_3_^−^–N treatment promoted the accumulation of flavonoids and organic acids and improved DPPH radical scavenging ability in fruits. Therefore, NO_3_^−^–N treatment caused poor taste and appearance, but the ability to scavenge reactive oxygen species was higher. In contrast, NH_4_^+^–N treatment resulted in better taste and nutritional quality. In addition, the fruit size, firmness, and color brightness decreased gradually as the season progressed, while soluble solid, sugar, anthocyanin, ellagic acid, flavonoid, and VC contents first increased and then decreased, reaching a maximum during the S2 or S3 terms. There was a gradual increase in antioxidant capacity as the season progressed. In production practice, NH_4_^+^–N application is recommended for blackberries when aiming to improve fruit size, firmness, taste, and nutritional quality. When targeting large, brightly colored, and high-firmness fruit, picking mature blackberry fruit in the early harvests is ideal, while harvesting fruit in the middle and late stages is beneficial for improving taste and fruit quality, as well as antioxidant capacity. 

## Figures and Tables

**Table 1:** Formula of Hoagland nutrient solution for each treatment.

Components	CK Concentration (mg/L)	NH_4_^+^–N Concentration (mg/L)	NO_3_^–^–N Concentration (mg/L)	Urea Concentration (mg/L)
(NH_4_)_2_SO_4_	0	991	0	0
CaCl_2_ ·2H_2_O	588	588	0	588
Ca(NO_3_)_2_ ·4H_2_O	0	0	945	0
NaNO_3_	0	0	595	0
CO(NH_2_)_2_	0	0	0	450.45
KCl	373	373	373	373
KH_2_PO_4_	136	136	136	136
MgSO_4_ ·7H_2_O	493	493	493	493
FeNaEDTA	36.7	36.7	36.7	36.7
KI	0.83	0.83	0.83	0.83
H_3_BO_3_	6.2	6.2	6.2	6.2
MnSO_4_ ·H_2_O	16.9	16.9	16.9	16.9
ZnSO_4_ ·7H_2_O	8.6	8.6	8.6	8.6
Na_2_ MoO_4_ ·2H_2_O	0.25	0.25	0.25	0.25
CuSO_4_ ·5H_2_O	0.025	0.025	0.025	0.025
CoCl_2_ ·6H_2_O	0.025	0.025	0.025	0.025

**Table 2:** Effects of different N treatments on the size, weight, and firmness of blackberry fruits.

Term	Treatment	Horizontal Diameter (mm)	Longitudinal Diameter (mm)	Weight (g)	Firmness (kg·cm^−2^)
	CK	23.40 ± 1.36 b	28.30 ± 1.24 b	7.57 ± 0.76 b	0.36 ± 0.03 c
	NH_4_^+^–N	25.59 ± 1.12 a	31.41 ± 1.06 a	9.31 ± 1.02 a	0.54 ± 0.05 a
S1	NO_3_^−^–N	23.31 ± 1.25 b	29.81 ± 1.15 ab	8.36 ± 0.86 ab	0.47 ± 0.04 b
	Urea	24.76 ± 1.06 ab	30.20 ± 0.60 a	8.70 ± 0.76 ab	0.52 ± 0.04 ab
	CK	20.88 ± 1.10 b	26.58 ± 2.21 c	6.72 ± 0.98 c	0.41 ± 0.05 b
	NH_4_^+^–N	24.76 ± 1.70 a	30.30 ± 1.29 a	10.17 ± 1.15 a	0.48 ± 0.04 a
S2	NO_3_^−^–N	23.46 ± 0.84 ab	27.81 ± 1.16 b	8.18 ± 0.64 b	0.44 ± 0.04 ab
	Urea	24.78 ± 1.45 a	28.54 ± 1.72 ab	9.60 ± 0.70 a	0.43 ± 0.05 ab
	CK	21.89 ± 1.56 c	25.60 ± 1.71 c	7.98 ± 1.46 b	0.34 ± 0.08 b
	NH_4_^+^–N	24.07 ± 1.61 a	30.68 ± 1.74 a	10.36 ± 0.64 a	0.41 ± 0.03 a
S3	NO_3_^−^–N	22.19 ± 1.51 bc	27.11 ± 2.03 c	7.94 ± 0.70 b	0.41 ± 0.05 a
	Urea	23.62 ± 1.53 ab	29.65 ± 2.16 b	9.58 ± 1.54 a	0.36 ± 0.04 ab
	CK	19.53 ± 1.79 c	24.30 ± 0.93 b	7.17 ± 1.33 ab	0.42 ± 0.04 b
	NH_4_^+^–N	22.35 ± 1.60 a	29.02 ± 2.03 a	8.07 ± 0.62 a	0.53 ± 0.07 a
S4	NO_3_^−^–N	20.86 ± 1.96 b	26.56 ± 2.25 b	6.69 ± 0.90 b	0.46 ± 0.08 b
	Urea	22.33 ± 1.74 a	27.19 ± 2.33 ab	7.71 ± 1.20 ab	0.42 ± 0.05 b
	S1	24.26 ± 1.48 A	29.93 ± 1.51 A	8.51 ± 1.04 A	0.47 ± 0.08 A
	S2	23.47 ± 1.69 B	28.31 ± 2.67 B	8.67 ± 1.60 A	0.44 ± 0.05 B
Term	S3	22.94 ± 1.83 B	28.26 ± 3.00 B	8.97 ± 1.62 A	0.38 ± 0.06 C
	S4	21.26 ± 1.83 C	27.27 ± 2.18 C	7.41 ± 1.14 B	0.37 ± 0.08 C
	Term	0.0003 ***	0.0073 **	0.0244 *	0.0192 *
*p*	Treatment	0.0027 **	0.0003 ***	0.0031 **	0.0112 *
	Interaction	0.2818	0.0034 **	0.0003 ***	0.000 ***

The data indicated are the means ± SDs (*n* = 20). The Duncan test was used to compare the means of all paired measurement values (*p* < 0.05). Different lowercase letters for the values in the same column indicate significant differences between the N forms at each time. Different capital letters for the values in the same column indicate significant differences among different times (* represents *p* < 0.05; ** represents *p* < 0.01; *** represents *p* < 0.001; no asterisk—statistically nonsignificant).

**Table 3:** Effects of different N treatments on the soluble solid and acid contents and color of blackberry fruits.

Term	Treatment	Soluble Solid Content	Acid Content (%)	L *	a *	b *
	CK	10.07 ± 0.06 b	1.54 ± 0.01 c	14.49 ± 2.53 c	0.14 ± 0.17 b	−3.19 ± 0.45 a
	NH_4_^+^–N	10.43 ± 0.06 a	1.54 ± 0.01 c	15.96 ± 2.01 b	0.27 ± 0.13 b	−3.13 ± 0.58 a
S1	NO_3_^−^–N	9.43 ± 0.21 c	1.91 ± 0.02 a	17.72 ± 2.11 a	0.15 ± 0.23 b	−3.26 ± 0.47 a
	Urea	10.37 ± 0.06 a	1.79 ± 0.01 b	17.44 ± 1.53 ab	0.34 ± 0.31 a	−3.28 ± 0.38 a
	CK	10.67 ± 0.06 d	1.69 ± 0.03 a	14.59 ± 1.18 b	0.28 ± 0.05 a	−3.25 ± 0.38 a
	NH_4_^+^–N	12.83 ± 0.21 a	1.26 ± 0.01 d	16.08 ± 1.66 a	0.26 ± 0.12 a	−2.95 ± 0.41 a
S2	NO_3_^−^–N	11.50 ± 0.10 c	1.46 ± 0.02 c	16.58 ± 1.58 a	0.36 ± 0.22 a	−2.29 ± 0.35 a
	Urea	11.80 ± 0.20 b	1.50 ± 0.01 b	17.29 ± 1.16 a	0.42 ± 0.12 a	−2.13 ± 0.23 a
	CK	11.13 ± 0.06 d	1.56 ± 0.02 b	11.66 ± 1.16 c	0.28 ± 0.06 a	−2.76 ± 0.41 a
	NH_4_^+^–N	13.13 ± 0.21 a	1.33 ± 0.01 c	15.37 ± 1.25 b	0.10 ± 0.12 a	−3.23 ± 0.41 a
S3	NO_3_^−^–N	11.93 ± 0.15 c	1.59 ± 0.03 b	15.97 ± 3.11 ab	0.19 ± 0.15 a	−3.22 ± 0.41 a
	Urea	12.47 ± 0.15 b	1.69 ± 0.01 a	17.24 ± 0.99 a	0.14 ± 0.21 a	−3.46 ± 0.32 a
	CK	10.17 ± 0.06 ab	1.66 ± 0.02 b	11.48 ± 1.56 b	0.10 ± 0.08 a	−2.80 ± 0.39 a
	NH_4_^+^–N	10.30 ± 0.10 a	1.48 ± 0.01 d	17.01 ± 1.25 a	0.28 ± 0.14 a	−3.13 ± 0.48 a
S4	NO_3_^−^–N	9.63 ± 0.06 c	1.71 ± 0.02 a	16.55 ± 1.74 a	0.21 ± 0.23 a	−3.20 ± 0.48 a
	Urea	9.97 ± 0.06 b	1.57 ± 0.01 c	17.02 ± 1.45 a	0.25 ± 0.12 a	−3.36 ± 0.31 a
	S1	10.08 ± 0.42 C	1.70 ± 0.02 A	16.40 ± 2.40 A	0.32 ± 0.38 A	−3.22 ± 0.46 B
	S2	11.70 ± 0.82 B	1.48 ± 0.01 D	16.63 ± 1.44 A	0.33 ± 0.36 A	−2.65 ± 0.83 B
Term	S3	12.17 ± 0.78 A	1.54 ± 0.01 C	15.06 ± 2.76 B	0.18 ± 0.20 A	−3.17 ± 0.47 B
	S4	10.02 ± 0.27 C	1.61 ± 0.03 B	15.52 ± 2.78 B	0.22 ± 0.21 A	−3.12 ± 0.46 B
	Term	0.0002 ***	0.0000 ***	0.3178	0.4403	0.1244
*p*	Treatment	0.0235 *	0.0399 *	0.0117 *	0.3592	0.7443
	Interaction	0.0000 ***	0.0000 ***	0.0000 ***	0.0043 **	0.0000 ***

The data indicated are the means ± SDs (n = 20). The Duncan test was used to compare the means of all paired measurement values (p < 0.05). Different lowercase letters for the values in the same column indicate significant differences between the N forms at each time. Different capital letters for the values in the same column indicate significant differences among different times (* represents *p* < 0.05; ** represents *p* < 0.01; *** represents *p* < 0.001; no asterisk—statistically nonsignificant).

**Table 4:** Eigenvalues and cumulative variance contribution rates of fourteen variances.

Component	Eigenvalues	Percent of Variance Explained/%	Cumulative Variance Contribution Rate/%
1	7.671	54.791	54.791
2	2.043	14.592	69.384
3	1.397	9.979	79.363
4	1.044	7.459	86.822
5	0.505	3.605	90.427
6	0.422	3.013	93.440
7	0.297	2.122	95.563
8	0.256	1.828	97.391
9	0.194	1.385	98.776
10	0.094	0.673	99.448
11	0.036	0.260	99.708
12	0.032	0.230	99.938
13	0.008	0.058	99.996
14	0.001	0.004	100.000

**Table 5:** Eigenvalues of each principal component.

Trait	PC1	PC2	PC3
FW	0.9 **	−0.058	−0.082
HD	0.118	−0.884 **	0.178
TSS	0.852 **	0.448	−0.022
Acid	−0.722 **	−0.174	−0.045
Fructose	0.897 **	0.19	−0.083
Glucose	0.862 **	0.399	−0.049
Sucrose	0.903 **	0.273	−0.168
Anthocyanin	0.881 **	−0.259	0.092
EA	0.88 **	−0.332	−0.013
Polyphenol	0.854 **	−0.103	0.22
Flavonoid	−0.298	0.367	−0.416
VC	0.553	−0.223	0.544
T-AOC	−0.057	0.53	0.775 **
DPPH-RSC	−0.749 **	0.322	0.439
Variance % Cumulative %	54.791 54.791	14.592 69.394	9.979 79.363

Note: ** represents eigenvalues that are significant, i.e., >0.60.

## Data Availability

The data generated for this study are available on request to the corresponding author.
